# High Rates of Hepatitis B and C and HIV Infections among Blood Donors in Cameroon: A Proposed Blood Screening Algorithm for Blood Donors in Resource-Limited Settings

**DOI:** 10.1155/2012/458372

**Published:** 2012-09-19

**Authors:** Florent Fouelifack Ymele, Basile Keugoung, Jeanne Hortense Fouedjio, Nadege Kouam, Sandrine Mendibi, Jacqueline Dongtsa Mabou

**Affiliations:** ^1^Yaoundé Central Hospital, P.O. Box 31186, Yaoundé, Cameroon; ^2^Research, Education and Health Development Associates Group (REHDAG), Dschang, Cameroon; ^3^Ministry of Public Health, Yaoundé, Cameroon; ^4^Faculty of Medicine and Biomedical Sciences, University of Yaoundé I, P.O. Box 1364, Yaoundé, Cameroon; ^5^Institute for Training and Demographic Research, P.O. Box 5644, Yaoundé, Cameroon; ^6^Yaoundé Central Hospital, Blood Bank Unit, P.O. Box 31186, Yaoundé, Cameroon

## Abstract

*Background*. Infections with human immunodeficiency virus (HIV), hepatitis B (HBV), and hepatitis C virus (HCV) are currently major public health problems. *Methods*. A retrospective study was conducted from January to June 2008 at the Blood Bank of the Central Hospital, Yaoundé (Cameroon). The objective was to study the prevalence of HIV, HBV, and HCV and their coinfections among blood donors. *Results*. A total of 4650 donors were identified, and the sex ratio (male/female) was 14/1. The median age of donors was 28 years (range: 16 to 69 years). Among blood donors, HBV, HIV, and HCV infection prevalences were 12.14%  (*n* = 565) , 4.44%  (*n* = 206), and 1.44%  (*n* = 67), respectively. Coinfection with HIV and HBV was observed among 0.77% donors, followed by hepatitis B and C co-infection (0.21%) and HIV and HCV coinfection (0.06%). Co-infection with HIV-HBV-HCV was encountered in 2 donors. The HIV, HBV, and HCV infections lead to a destruction of one out of six sets of blood collected. *Conclusion*. There is a need to review policies for blood collection from donors, by modifying the algorithm of blood donors testing. Pretesting potential donors using rapid tests could help to avoid collection and destruction of (infected) blood.

## 1. Introduction

Infections due to the human immunodeficiency virus (HIV), hepatitis B virus (HBV), and hepatitis C virus (HCV) are major public health problems worldwide [1]. In sub-Saharan Africa, these infections are frequent among the general population and blood donors [2, 3]. In 2010, 68% of the 34million of people infected with HIV were living in sub-Saharan Africa [4]. Between 1983 and 2001, the Central African region had the highest HCV prevalence worldwide with a prevalence of 6% and this prevalence was 13.8% in Cameroon [5]. The seroprevalence of hepatitis B S antigen among blood donors in Cameroon was 10.7% in 2003 [2].

The HIV, HBV, and HCV coinfections are severe and frequent in sub-Saharan Africa. In a systematic review, the mean prevalence rates of HBV and HCV among HIV-positive people were 15% and 7%, respectively [6]. Indeed, these viruses have the same mode of transmission through sexual intercourse, mother-to-child, and blood transfusion. Furthermore, their coinfection has more negative effects. The influence of HIV on HBV is characterized by a more frequent evolution towards chronicity, an increased viral replication rate, a viral reactivation leading to fibrosing cholestatic hepatitis, and an increased progression towards fibrosis and liver cirrhosis [7–9]. On the other hand, the HBV infection aggravates the progression towards AIDS and an increased in vitro replication of HIV [10]. Unfortunately, access to treatment against these viruses is not always accessible for most people in developing countries. Even though significant improvement has been achieved, in 2010, only 41% of HIV positive eligible people had access to antiretroviral therapy in sub-Saharan Africa [4]. Therefore, screening for these three viruses is imperative before any blood transfusion.

Studies have shown that in sub-Saharan Africa, international models and standards for organizing blood banks and blood donation were not affordable and might even be unsustainable. Consequently, these models and standards should be adapted to resource poor environments [11]. Even though improvement has been achieved, many challenges remain unsolved to ensure blood safety especially in rural areas [12]. Tagny and colleagues [13] revealed that in sub-Saharan Africa, there were several problems undermining the blood safety including the low implementation of national policies for transfusion, organizational dysfunctions, inadequate financing, and the lack of adequate blood screening equipment. Despite these shortcomings, measures are urgently needed to ensure blood safety and to avoid the spread of infectious agents and the deterioration of blood donors and of blood recipients' health. Therefore, the safety of not only blood recipients but also of blood donors should guide the blood safety procedures.

Few studies have been carried out in recent years on the coinfections with HIV, HBV, and HCV among blood donors in Cameroon, but none has analyzed the procedures used for ensuring blood safety. Knowing the prevalence of these viruses among blood donors will underpin the extent to which these infections are present in Cameroon. Additionally, identifying safety issues in blood donation procedures will guide health actors to urgently develop and implement efficient strategies for ensuring blood safety.

Therefore, the objectives of this study are to determine the prevalence of HIV, HBV, and HCV and their coinfections among blood donors at the Yaounde Central Hospital and to propose an efficient screening algorithm of blood donors for resources limited settings. 

## 2. Material and Methods 

We carried out a retrospective study from January to June 2008 at the Yaounde Central Hospital. Data was collected from the registers of the blood bank and was filled in a data collecting form. Observation was used to describe the blood donation procedures at the blood bank. All blood donors registered at the blood bank during the study period were included in the study.

Data retrieved included the demographic characteristics of donors: age, sex, residence, and the results of HIV, HBV, and HCV serologies. 

Data on blood donors and the results of all performed tests on their blood were entered on Microsoft Excel 2007 spreadsheets by one investigator then controlled by a second investigator. Data was analyzed using the SPSS 19 software. Bivariate analysis was used to find correlation between characteristics of donors and studied infections. The following indicators were assessed: (i) the distribution of blood donors per age and sex; (ii) the number and ratio of persons infected by HIV, HBV, and HCV viruses and of persons coinfected by two or three viruses; lastly (iii) the ratio of blood donors infected by HIV, HBV, and HCV by age group. Tables and figures were constructed.

Medians, means, and ratios were calculated. The chi-square test was used to compare means. The difference was considered statistically significant for *P* value < 0.05. All data was aggregated to ensure confidentiality. We did not meet or interact with donors or patients.

## 3. Results

Of the 4650 donors recruited for the study, the sex of 4645 (99.9%) donors was available. The age of 4630 (99.6%) donors was available while HIV, HBV, HCV, and syphilis tests were conducted in 4641 (99.8%) donors. 

### 3.1. Procedures for Blood Donation and Safety Procedures

The Yaounde Central Hospital Blood Bank is the largest and the most used in Cameroon. Current procedures of screening blood donors consist of two types of blood donations—voluntary or benevolent donation and family donation. Benevolent donations represent less than 25% of all blood donations in Cameroon in general [4]. In this case, blood is collected during public sensitization campaigns from voluntary donors. 

Regarding family blood donation, a family that has a patient in need of blood transfusion brings its blood donors found within the family or not. Two donors are required by the hospital for each set of no infected blood that will be provided to the patient. Counselling is not done to the donor and in general, donors do not return to the blood bank to have information about the results of the tests performed on their collected blood.

In both cases, data on the blood donor are registered and include name, age, sex, profession, and telephone number. Blood is collected from donors and tests are carried out later on to check if the blood is safe and to categorize the blood group. These tests consist of blood group and Rhesus, the haemoglobin electrophoresis, and the screening of infections using HIV serology, HBV antigen S test, HCV serology, and syphilis serology (TPHA and VDRL). If one or more of these infection tests is positive, the collected blood set is destroyed. 

### 3.2. Demographic Characteristics

**Figure 1:**
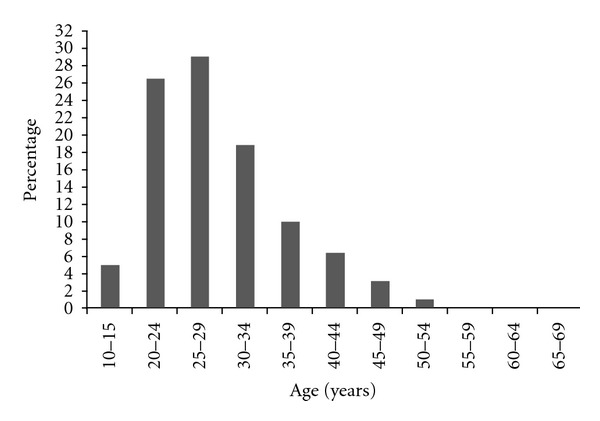
Distribution of blood donors by age group. Each row represents the proportion of the number of donors in the age group among the 4645 blood donors.

Out of the 4645 blood donors, 4337 (93.37%) donors were males while 308 (6.67%) donors were females giving a male-to-female sex ratio of 14 : 1. The age of blood donors varied from 16 to 69 years with a median age of 28 years. The majority of donors (55%) had between 20 and 29 years of age (see Figure 1).

### 3.3. Seroprevalence of HIV, HBV, and HCV and Their Coinfections in Blood Donors

The results of the laboratory tests were available for 4644 (99.98%) blood donors, of which 564(12.14%) were infected with the HBV, 206 (4.44%) with the HIV, and 67 (1.46%) with the HCV (see Table 1). HBV infection was most frequent in men (*P* = 0.02). A total of 837 (18%) sets of blood were destroyed due to infection by at least one of the three viruses.

Coinfections were not very common in blood donors. A total of 36 (0.78%) cases of HIV and HBV coinfections were recorded, followed by HBV-HCV coinfection with a prevalence of 0.22% and a prevalence of 0.06% for HIV and HCV coinfection. The coinfection with the three viruses was 0.04% among blood donors (see Table 2).

### 3.4. Cross-Distributions between Ages and Infections

**Figure 2:**
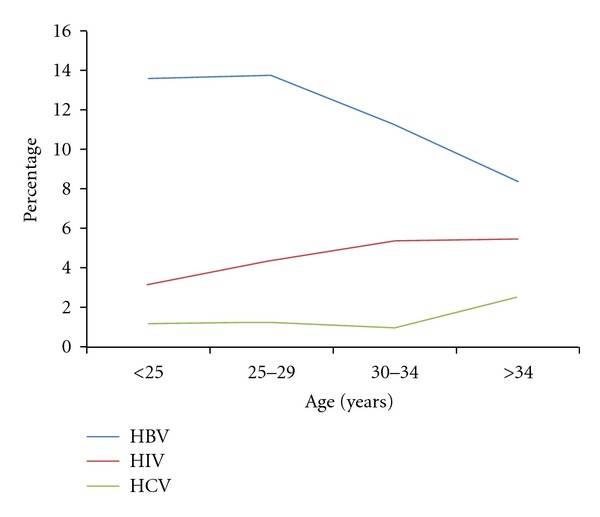
Prevalence of HIV, HBV, and HCV infections in blood donors according to age. The proportion of blood donors with a positive test was calculated over the total number of blood donors in the age group.

HIV, HBV, and HCV infections were found in all age groups (see Figure 2). The prevalence of HIV and HCV increased with the age of the donors. On the contrary, HBV infection prevalence decreased with the age of the donors. 

## 4. Discussion

The seroprevalence of HIV infection in blood donors (4.43%) was lower than the HIV seroprevalence of 5.5% found in the adult population between 15 and 49 years in 2004 during the National Demographic Survey [14]. This could be explained by the fact that families search for “physically healthy” blood donors. 

Regarding HBV infection, the prevalence seems to be increasing with time. In 1993, Ndumbe and Skalsky [15] found a HBV prevalence of 6.7% while Mbanya et al. [2] recorded a prevalence of 10.7% in Cameroon in 2001, compared to 12.14% in our study. They conducted their study among blood donors in a hospital at Yaoundé but they had a sample size (252 blood donors) 18 times much lower than in our study. In 2006, hepatitis B vaccination was introduced in the Expanded Program of Immunization of Cameroon but only for children below one year of age. Other studies have shown the high endemicity of HBV in other sub-Saharan African countries but with various prevalences [16–21]. Blood transfusion is the major route of transmission of HCV. In a study carried out in Nigeria on blood donors and sickle cell patients, the prevalence of HCV was recorded at 14% by Mutimer et al. [22]. In developed countries, the risk of HCV infection through transfusion is low and was estimated at 1 on 3000 transfusions due to its low prevalence among the general population and the adequate checkup [23]. 

Regarding coinfections, the prevalences of HIV-HBV and HIV-HCV were much lower than results obtained by Laurent et al. [24] and Mbanya et al. [2] in 2001 at Yaounde. Indeed, Laurent et al. found among HIV-positive patients, 8.3% and 12.4% coinfections with HBV and HCV, respectively. But these results were obtained from 169 HIV-positive people eligible for antiretroviral therapy, thus at a more advanced stage of the HIV infection.

The presence of HIV, HBV, and HCV coinfections increases the risk of disease in blood donors. Moreover, collection of blood from an infected donor could increase the progression to a symptomatic or disease stage. In fact, the loss of 500 mL of blood reduces the haemoglobin level by about 1 g as well as leucocytes and antibodies of the donor. De Cock and colleagues argued that some approaches to fight against HIV/AIDS and other infectious diseases were often poorly adapted to adequately address the disease [25].

The HIV/AIDS National Control Programme is in charge of HIV/AIDS and other sexually transmitted diseases in Cameroon. However, it mainly focuses on HIV/AIDS. This could be explained by the HIV/AIDS “exceptionalism” but neglecting other sexually transmitted infections such as HBV and HVC could lead to the spread of both these diseases and even HIV. Secondly, processing infection screening tests in parallel and on sets of blood already collected leads to wastage of screening tests and to a loss of blood collected from infected donors. 

**Figure 3:**
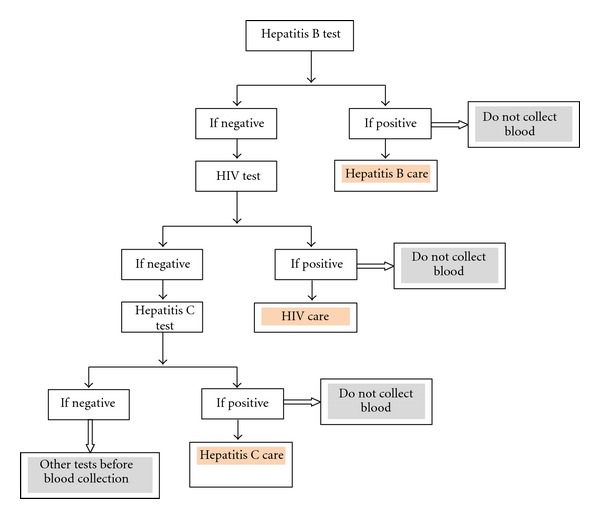
New algorithm for blood donors infections screening.

Consequently, we proposed new strategies for improving efficiency and management of blood donors in resource-limited settings and ensuring blood safety (Figure 3). Firstly, the screening tests should be performed in series. The next test will only be carried out if the previous one is negative. The order in which the tests should be processed will be based on the prevalence of the infections found in the country starting from the most prevalent infection and finishing with the least frequent one. With regard to the Cameroon context, the HBV test will be performed first, followed by the HIV test, and finally the HCV test. Other tests such as syphilis test can be performed depending on the local epidemiology. This strategy is more efficient in the sense that it avoids the wastage of materials, reagents, and time and is done on a small sample of blood.

Secondly, blood will be collected only if all the tests are negative, avoiding blood collection from infected donors. This has the advantages of saving time and reducing workload for processing all the tests. The time saved should be used to improve counselling of infected donors, and prepare them for future clinical followup and care.

The implementation of this algorithm requires a new approach towards blood donation. This approach includes counselling of blood donors, carrying out rapid tests and communicating the results to donors, and putting in place strategies for the care of infected blood donors. This strategy even goes beyond efficiency and encompasses moral and ethical considerations to offer quality health care to people willing to save other lives through blood donation while being themselves in urgent need of care. 

## 5. Conclusion

HIV, HBV, and HCV infections are frequent among blood donors in Cameroon. Collecting sets of blood and carrying out screening tests later are not efficient. Serial tests should be processed on blood samples using rapid tests, and sets of blood should be collected only from donors with negative tests. Lastly, strategies should be put in place to take care of infected blood donors.

## Figures and Tables

**Table 1:** Prevalence of HIV, HBV, and HCV among blood donors.

Sex	HIV test			HBV test			HCV test			Total positive
	Positive (%)	Negative (%)	Total (%)	Positive (%)	Negative (%)	Total (%)	Positive (%)	Negative (%)	Total (%)
Male	192 (4.43)	4144 (85.57)	4336 (100)	544 (12.55)	3792 (87.45)	4336 (100)	65 (1.49)	4271 (98.50)	4336 (100)	800 (18.5)
Female	14 (4.55)	294 (95.45)	308 (100)	20 (6.49)	288 (93.51)	308 (100)	2 (0.65)	306 (99.35)	308 (100)	37 (12)

Total	**206 (4.44)**	4438 (95.56)	4644 (100)	**564 (12.14)**	4080 (87.86)	4644 (100)	**67 (1.46)**	4577 (98.56)	4644 (100)	837 (18)

*P* value			0.923			0.02			0.227	

In Table 1, the results of HIV, HBV, and HCV tests are presented by blood donor sex. The proportion of blood donors with a positive test was compared by sex group using the chi-square test.

**Table 2:** Seroprevalence of HIV, HBV, and HCV coinfections according to sex among blood donors.

Sex	Co-infection VIH-VHB			Co-infection VIH-VHC			Co-infection VHB-VHC			Co-infection VIH-VHB-VHC		
	Positive (%)	Negative (%)	Total (%)	Positive (%)	Negative (%)	Total (%)	Positive (%)	Negative (%)	Total (%)	Positive (%)	Negative (%)	Total (%)
Male	32 (0.74)	4303 (99.26)	4335 (100)	3 (0.07)	4333 (99.93)	4336 (100)	9 (0.21)	4326 (99.79)	4336 (100)	2 (0.05)	4333 (99.95)	4335 (100)
Female	4 (1.30)	304 (98.70)	308 (100)	0 (0.00)	308 (100.00)	308 (100)	1 (0.32)	307 (99.68)	308 (100)	0 (0.00)	308 (100.00)	308 (100)

Total	36 (0.78)	4607 (99.22)	4643 (100)	3 (0.06)	4644 (99.94)	4644 (100)	10 (0.22)	4633 (99.78)	4644 (100)	2 (0.04)	4641 (99.96)	4643 (100)

*P* value			0.279			0.644			0.668			0.706

In Table 2, the results of coinfections with HIV, HBV, and HCV among blood donors are presented by blood donor sex. The proportion of blood donors with a positive coinfection was compared by sex group using the chi-square test.
